# Dental Management of a 14-Year-Old with Cockayne Syndrome under General Anesthesia

**DOI:** 10.1155/2014/925258

**Published:** 2014-12-10

**Authors:** Divya Gaddam, Mukesh Singh Thakur, Niranjani Krothapalli, Saujanya Kaniti

**Affiliations:** ^1^Department of Pediatric Dentistry, Mamata Dental College, Khammam, Telangana 507002, India; ^2^Department of Pediatric Dentistry, HKDET Dental College, Humnabad, Karnataka 585330, India; ^3^Department of Pediatric Dentistry, St. Joseph Dental College, Eluru, Andhra Pradesh 534003, India; ^4^Department of Pediatric Dentistry, Sree Sai Dental College, Srikakulam, Andhra Pradesh 532001, India

## Abstract

Cockayne's syndrome is a rare, autosomal recessive disorder characterized clinically by cachectic dwarfism, cutaneous photosensitivity, loss of adipose tissue, mental retardation, skeletal and neurological abnormalities, and pigmentary degeneration of the retina. Dental caries is a common finding. Dental rehabilitation of a 14-year-old male with Cockayne's syndrome is presented.

## 1. Introduction

Cockayne's syndrome (CS) is a rare, autosomal recessive disorder first reported in siblings in 1936 (Cockayne's 1936; Otsuka and Robins 1985) [1]. This syndrome occurs with a frequency of 1/1,00,000 in live births and can be caused by mutations of two genes, the Cockayne Syndrome Type I (CKN1) or Excision-Repair Cross-Complementing Group 8 (ERCC8) and the Excision-Repair Cross Complementing Group 6 (ERCC6), located on chromosomes 5 and 10q11, respectively [2]. It has also been associated with mutations in XPB (Xeroderma pigmentosum B) gene, XPD (Xeroderma pigmentosum D) gene, and XPG (Xeroderma pigmentosum G) gene. The CS clinical findings are growth failure, premature aging, short stature, cachectic aspect, disproportionately long limbs, kyphosis, microcephaly, and sparse hair. The findings also show a lack of subcutaneous facial fat (particularly of the cheeks), prominence of the facial bones, sunken eyes, a thin and beaklike nose, and large ears, which all give the patient a “birdlike” appearance. They also presented with complications like delayed psychomotor skills, mental retardation, liver and spleen enlargement, renal disease, and hypertension [3]. Craniofacial anomalies and dysmorphism like microcephaly (a small head) with retrognathia (a distally placed mandible), high arched palate, atrophy of the alveolar process, condylar dysplasia, absence of some permanent teeth, and short roots have been described. Orodental features like delayed deciduous tooth eruption, malocclusion, and absent/hypoplastic teeth were also mentioned by Nance and Berry but with no detailed analysis. Dental anomalies including dental caries are considered to be a minor diagnostic feature in this milestone paper together with photosensitivity, progressive retinitis pigmentosa, and deafness [4]. CS Type A (Type I) is defined as the classical milder form of the syndrome whereas CS Type B (Type II) is the early onset severe form, which can lead to early death. Cerebrooculofacioskeletal (COFS) syndrome is a more severe prenatal form of CS with clinical expression similar to Type II. CS Type III has mild symptoms and onset in late childhood. Different severity groups have however been described and renamed recently: severe, moderate, and mild CS. Mean age of death is 5, 16, and 30 years in these groups, respectively [5]. In this report we aimed to present the dental treatment of a CS case done under general anesthesia because of the multisystemic anomalies.

## 2. Case Report

In the present case, a 14 year old male patient with negative family history was born weighing 2.9 kgs. The parents gave a positive history of consanguinity and first became concerned about the child at approximate age of 3 months because his head did not appear to be growing. The patient continued to experience a deceleration in growth rate in height, weight, and head circumference. He rolled over at 3 months but was never able to crawl, walk, or sit without support.

The diagnosis of CS was made by the pediatricians of ASRAM Medical College, Eluru, Andhra Pradesh, at the age of 4 years. The patient was strikingly emaciated, nonverbal, and nonambulatory. He appeared to have cachectic habitus, kyphosis, sunken eyes, a thin and beaklike nose, lack of subcutaneous cheeks fat, and large ears, giving the patient a “birdlike” appearance. He had motor and mental retardation. Other physical findings include thin atrophic hyperpigmented skin with sensitivity to sunlight, limitation of joint mobility of the legs and lower back, scoliosis, decreased sweating and tearing, sparse hair, low set ears, cool hands and feet, and sometimes cyanotic, cataracts, and premature calcifications especially of the central nervous system but not renal disease. Medical history revealed that he is undergoing treatment for epilepsy, pneumonia, and tuberculosis and he also presented with nasogastric feeding and regurgitation or vomiting after meals.

**Figure 1:**
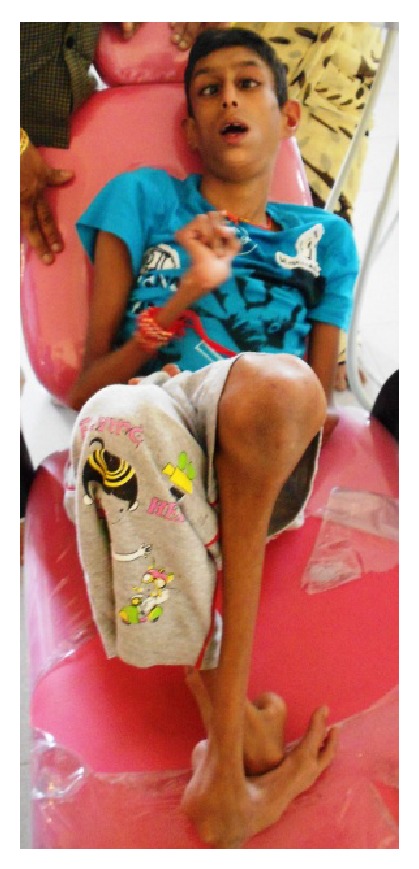
14-year-old patient. Note is made of Sunken eyes, “birdlike” appearance, and lack of adipose tissue.

**Figure 2:**
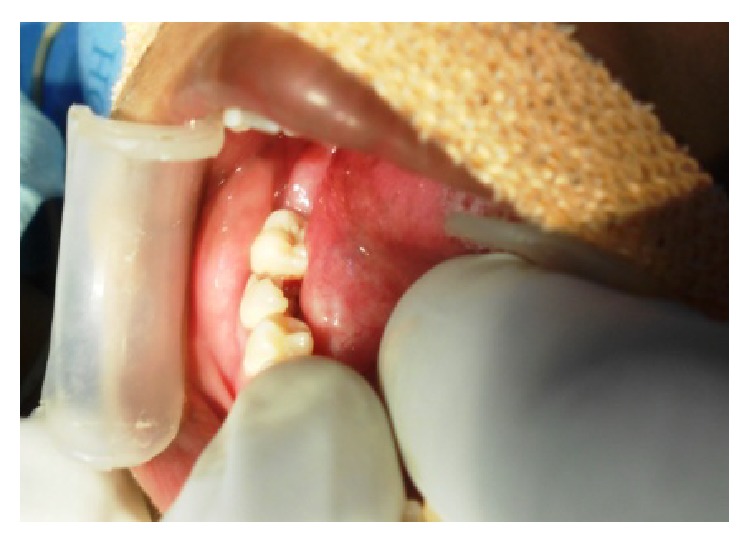
Grossly decay irt 46.

**Figure 3:**
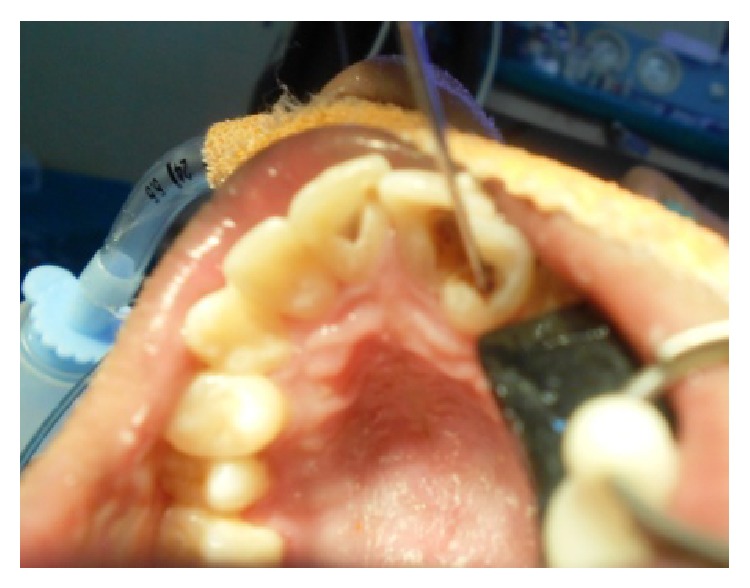
Deep dental caries irt 11 and fused crowns 21, 22.

The patient's parents reported to our department at the age of 14 with a chief complaint of pain in lower right back tooth region (Figure 1). An examination limited by poor cooperation revealed multiple gross carious lesions with poor oral hygiene. Intraoral findings showed deeply arched palate and the temporomandibular joint demonstrated a characteristic restriction of motion with teeth present 11, 12, 13, 14, 15, 16, 17, 21, 22, 23, 24, 25, 26, 27, 31, 32, 33, 34, 35, 36, 37, 41, 42, 43, 44, 45, 46, and 47. Among these 46 has been grossly decayed (Figure 2) and deep dentinal caries irt 11, fused crowns irt 21, 22 (Figure 3), cervical dentinal caries irt 14, 15, 24, 25 has been observed. Views obtained by direct laryngoscope scored grade 2 (half of the glottis seen, at worst only the posterior tip of arytenoids is seen) according to Cormack-Lehane classification (1984) which shows likelihood of difficult intubation. After placement of appropriate monitors in the operating room, anesthesia was given using Ketamine and Propofol 2 mg/kg. We reverted to nitrous oxide gas instead of midazolam because of the effectiveness of the gas. Using nitrous oxide gas as a sedative agent, the dentist-anaesthetist could secure an intravenous route using a 24-gauge catheter and an infusion of dextrose 5% in 0.2% saline was begun. To prevent bradycardia atropine 0.01 mg/kg was given.

**Figure 4:**
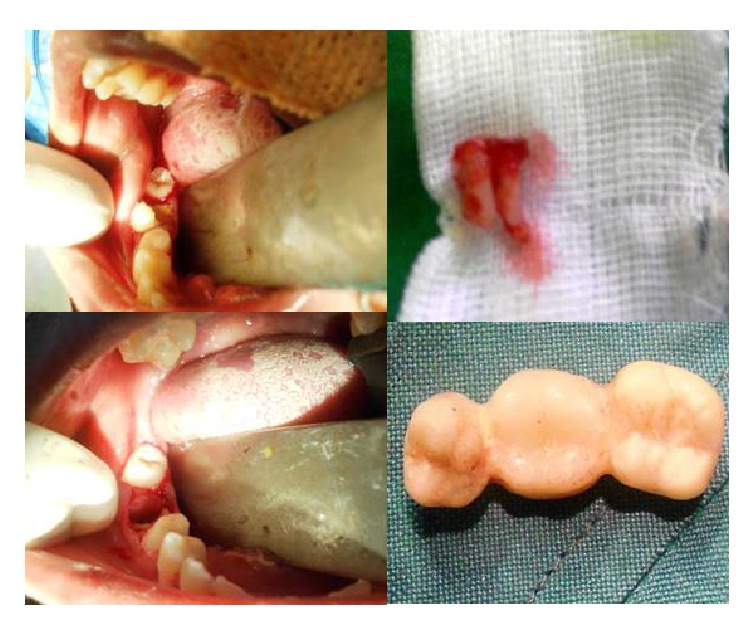
Tooth preparation of 45, 47 followed by extraction of 46 and acrylic temporization irt 45, 46, 47.

**Figure 5:**
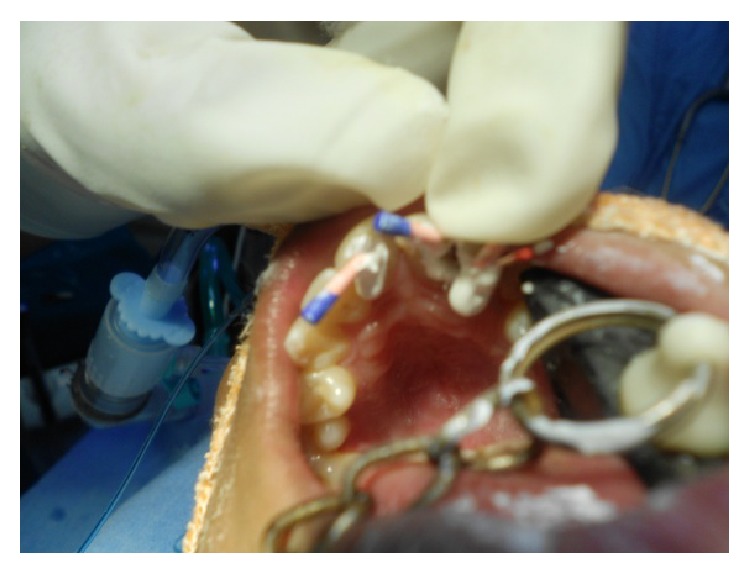
Root canal treatment irt 11, 21, 23.

**Figure 6:**
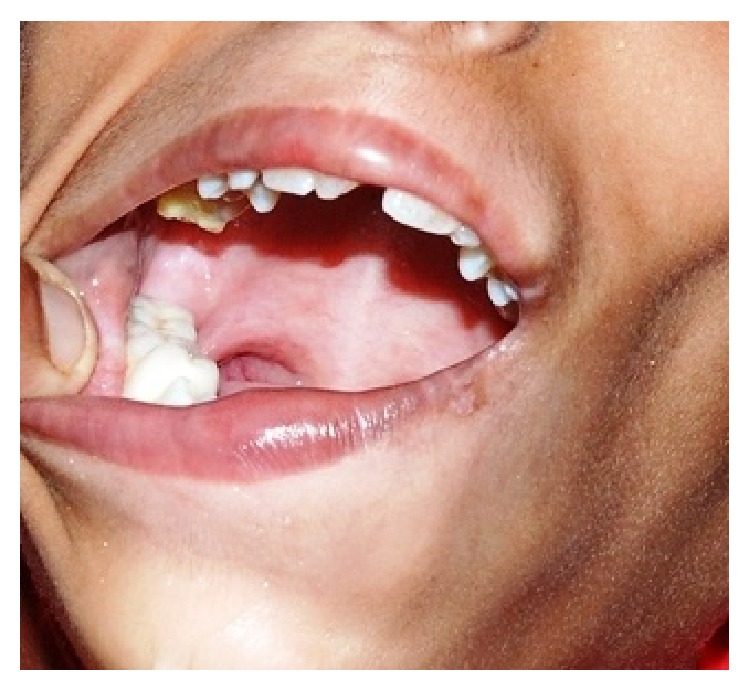
Fixed ceramic prosthesis irt 45, 46, 47.

Treatment rendered to the patient under nasotracheal intubation general anesthesia was oral prophylaxis, tooth preparation of 45, 47 followed by extraction of 46 and acrylic temporization irt 45, 46, 47 (Figure 4), root canal treatment irt 11, 21, 23 (Figure 5), and glass ionomer restorations irt 14, 15, 24, 25 and finally fluoride varnish was applied. Patient was recalled after 1 week for insertion of fixed ceramic prosthesis irt 45, 46, 47 (Figure 6).

## 3. Discussion

The clinical findings in our patient permitted us to establish a diagnosis of CS. As it was reported by Neil and Dingwall in 1950, premature aging seen in our patient is associated with CS and may be differentiated from progeria by the ocular anomalies and the cutaneous photosensitivity [6]. Mental deterioration is progressive and the dwarfism becomes obvious at this time. A characteristic facies develops, resulting in a thin prominent nose, prognathism, sunken eyes, and a lack of subcutaneous fat. Other major neurological abnormalities include sensorineural hearing loss, ataxia, spasticity, myoclonus, and gait disturbance; similar characteristic findings were noticed in the present case [6]. The usual oral findings are delayed deciduous teeth eruption, oligodontia, short roots, more incidence of caries, a deep palate, atrophy of the alveolar processes, mandibular prognathism, and condylar hypoplasia. We noticed macrodontia, fused incisors, and high arched palate in the present case [7]. Woodridge et al in 1996 reported difficult airway and intubation management and increased risk of gastric, later cachexia and accelerated aging issues as the most commonly encountered anesthetic problems in CS patients [8]. In addition to the above reasons, restricted temporomandibular joint movements and mouth opening made to opt for nasotracheal intubation general anesthesia in the present case. Although no cure for CS is imminent, it may be preventable. The syndrome can be diagnosed prenatally by examining amniotic cells cultured in vitro. The prenatal test can be carried out two or four days after culture of sufficient cells. An autoradiographic procedure can be used with a few hundred cells; therefore, it should be possible to obtain accurate results in two weeks (Lehman et al. 1985) [1].

## 4. Conclusion

Craniofacial and oral anomalies and dental caries are common in the CS. Although life expectancy is relatively short for these individuals, the pediatric dentist plays a significant role in managing the CS patient. Early dental evaluation and parental counseling have the utmost significance. Preventive dental regimens must be individually designed and implemented because of reduced mandibular motion. Dietary counseling is extremely important because of a propensity for dental caries and low weight. Frequent examinations and emphasis on preventing dental disease must be stressed to the parents because of the difficulty in providing restorative care. Appropriate and safe dental care for patients with CS can be rendered after medical consultation.
